# Bacterial communities during composting cultivation of oyster mushroom *Pleurotus floridanus* using broken eggs as the nitrogen source and study of agronomic and nutritional properties

**DOI:** 10.3389/fmicb.2023.1274032

**Published:** 2024-01-12

**Authors:** Jun Wei, Yue-Xin Wang, Ti-Kun Guan, Qiu-Ying Wang, Jiao Zhang, Jia-Yan Zhang, Jian-Li Wang, Qing-Jun Chen, Guo-Qing Zhang

**Affiliations:** College of Plant Science and Technology, Beijing Key Laboratory for Agricultural Application and New Technique, Beijing University of Agriculture, Beijing, China

**Keywords:** *Pleurotus floridanus*, broken eggs, composting cultivation, nutritional properties, bacterial communities

## Abstract

**Introduction:**

Broken eggs are a byproduct of the poultry industry and a potential nitrogen source for mushroom cultivation. However, its feasibility needs to be evaluated experimentally.

**Methods:**

In this study, a series of different addition amounts (0, 1.8, 3.6, 5.3 and 8.5%, w/w) of broken egg mixture (BEM) were applied in the composting cultivation process of oyster mushroom. The physicochemical properties and bacterial communities of composting substrate, and agronomic and nutritional properties of fruiting bodies were determined.

**Results and discussion:**

The results showed that the BEM addition significantly (*P* < 0.05) increased the total nitrogen content in the composted substrate, and the contents of crude protein, total amino acids and essential amino acids of mushrooms. The P3 treatment (initial C/N of 26:1) showed the highest biological efficiency (BE) of 100.19% and a low contamination rate (CR) of 7.00%, while the higher dosage of BEM (P4 and P5) led to a sharp decrease in BE and a sharp increase in CR. High throughput sequencing revealed that the addition of BEM significantly (*P* < 0.05) changed the bacterial communities in the substrate at the beginning of composting. *Streptococcus* and *Lactococcus* were predominant bacterial genera in BEM treatments at the beginning stage of composting, while *Acinetobacter* became predominant at the ending stage. The co-occurrence network analysis showed that the P3 treatment demonstrated a much more complex bacterial community. The structural equation model analysis indicated that the addition of BEM affected the bacterial communities and nitrogen metabolism during composting, which further affected agronomic and nutritional properties of oyster mushrooms. An appropriate amount of BEM combined with composting processes can significantly improve the yield and quality of oyster mushroom, providing a new way for efficient utilization of BEM.

## Introduction

Poultry eggs are a high-quality protein resource and one of the most important animal protein sources for humans (Tang et al., 2022). According to the data of the National Bureau of Statistics, the annual output of poultry eggs in China reached 34.68 million tons in 2020. However, due to the fragility of eggshells, broken eggshells have brought huge economic losses to the egg industry in the production, packaging, storing, transporting, and selling processes (Ma Y. et al., 2020). Approximately 10% of the poultry egg production is lost due to breakage of eggshells, which not only leads to huge economic losses, but also brings about the problem of using broken eggs (Sah et al., 2018; Jiang et al., 2021). Moreover, the broken eggs can serve as a potential high-quality nitrogen source for mushroom cultivation, especially for oyster mushrooms.

Oyster mushroom species, belonging to the genus *Pleurotus*, are one of the most cultivated and consumed mushrooms in the world, accounting for more than 16% of global annual production of edible mushroom (Wan Mahari et al., 2020). The oyster mushrooms have become world popular foods due to their delicious taste, rich nutrition and abundant biological activities (Correa et al., 2016; Rizzo et al., 2021). They are rich in protein and dietary fiber, and low in calories. Moreover, massive researches reveal that mushroom polysaccharides and glycoproteins demonstrate antitumor, antioxidant, anti-inflammatory, immunostimulatory, antidiabetic, anti-hyperlipidemia, hepatoprotective and detoxicating properties (Correa et al., 2016; Rodrigues Barbosa et al., 2020; Jacinto-Azevedo et al., 2021; Rizzo et al., 2021; Koutrotsios et al., 2022).

Oyster mushrooms are efficient lignocellulose decomposing fungi, which can be cultivated using various agricultural and forestry waste (Bellettini et al., 2019; Wan Mahari et al., 2020; Liu et al., 2022). The composting cultivation has become very popular in China, in which the raw materials undergo a short-term composting process (about 5–10 days) before pasteurization (or not) and mushroom spawning processes (Kong et al., 2020; Guo et al., 2021). In the process, the composted substrate is directly bagged and inoculated with spawns, or undergoes a short-term steam pasteurization process (100°C, about 1–4 h) before inoculation (Liu et al., 2022; Yang et al., 2022). The non- or short-term pasteurization of compost leads to the survival of microorganisms (especially bacterial species) in the composted substrate, which play important roles in improving the mushroom yield and reducing the CR (Kong et al., 2020; Yang et al., 2022). At the same time, different raw materials and cultivation techniques also lead to differences in the nutritional properties of fruiting bodies. Previous studies revealed that the crude protein and *β*-glucan contents of *Pleurotus ostreatus* grown on oil palm by-product formulated substrates were significantly higher than those grown on the control (Aubrey et al., 2022, 2023).

The nitrogen source is one of the most important factors for mushroom cultivation. It can affect the enzyme activities of mycelia, as well as the yield and quality of mushrooms. Agricultural by-products such as wheat bran and soybean meal are often used as nitrogen sources for oyster mushroom cultivation, while there is relatively little research on using poultry by-products as nitrogen sources (Bellettini et al., 2019; Wan Mahari et al., 2020). In addition, a proper amount of high-quality nitrogen source can improve the yield and quality of fruiting bodies (Bellettini et al., 2019; Kumla et al., 2020). However, few studies have reported the effects and mechanisms of composting microorganisms on the nutritional properties of oyster mushroom cultivated with the composted substrate. It is necessary to analyze the microbial communities in the composted substrates and their correlations with the nutritional properties of fruiting bodies. The aim of this study was to evaluate the feasibility and optimum amounts of broken eggs as the nitrogen source during the composting cultivation of oyster mushrooms, bacterial communities in the compost and their effects on the agronomic and nutritional properties. The results will also provide a sustainable approach for the application of broken eggs.

## Materials and methods

### Materials

The strain of oyster mushroom used was *Pleurotus floridanus* “Heiping 6,” which was collected in Laboratory of Edible and Medicinal Fungi, Beijing University of Agriculture (BUA, Beijing, China). The stock culture and grain spawns were prepared using the potato dextrose agar and wheat grain medium, respectively and cultured at 25 ± 2°C. Peach sawdust (2–20 × 2–3 mm) was crushed using naturally dried peach branches which were collected from Dahuashan Town (Beijing, China). The broken egg mixture (BEM), consisting of egg liquid, eggshells and water, was donated by a local layer farm. The corncob, wheat bran and lime were purchased from local markets. The physicochemical properties of raw materials were determined as described by Zou et al. (2020) and showed in Supplementary Table S1.

### Composting design and sampling

The composting formulas were determined based on the initial C/N ratio of substrates of approximately 20:1–30:1 and listed in Table 1 (Wan Mahari et al., 2020). The BEM treatments P1–P5 were added 5.60–28.00 kg BEM as the nitrogen source. A common formula using wheat bran as the nitrogen source was treated as the blank control treatment (CK) (Yang et al., 2022). Three parallel composting experiments were performed in the Beijing Science and Technology Backyard No. 38, Dahuashan Town, Beijing, China. The raw materials were well-mixed and adjusted the initial moisture content (MC) to approximately 60–65%, and then piled into trapezoidal piles as described by Yang et al. (2022). According to the actual production of farmers and our preliminary studies, the short-term composting process lasted for 3 days, and the compost was turned at the 2nd and 3rd day (Guo et al., 2021). Samples were collected at the beginning and ending of composting (numbered as BM and CP, respectively), and stored at – 80°C or 4°C for further analyses. Nine points random sampling method was adopted for sampling, and three samples were randomly collected at the top, middle, and bottom depths of the composting pile for mixing as one sample (Guo et al., 2021).

**Table 1:** Composting formulas and their C/N ratio.

Formula	Peach sawdust (kg)	Corncob (kg)	Broken egg mixture (kg)	Wheat bran (kg)	C/N
CK	210	90	0	50	32:1
P1	210	90	5.60	0	36:1
P2	210	90	11.20	0	33:1
P3	210	90	16.80	0	26:1
P4	210	90	22.40	0	24:1
P5	210	90	28.00	0	22:1

### Mushroom cultivation

After the short-term composting, the composted substrate was bagged (22 × 48 mm, approximately 2.0 kg fresh weight each), followed by a steam pasteurization at 100°C for 4 h (Wan Mahari et al., 2020; Yang et al., 2022). Each treatment contained 300 cultivation bags, which was randomly selected and equally divided into three parallel groups (100 bags each). After cooling down to room temperature, the cultivation bags were inoculated using the spawns (2%, v/v) and incubated in the dark at 25 ± 2°C until the mushroom mycelia fully colonized the substrate. Subsequently, the matured cultivation bags were moved into a greenhouse for the fruiting management and harvest (Yang et al., 2022). The fruiting bodies were collected for three flushes.

### Physicochemical analysis

The physicochemical properties of the composted substrate were determined, including the pH, electrical conductivity (EC), the content of moisture (MC), ash, organic matter (OM), total carbon (TC) and total nitrogen (TN), and the C/N ratio, followed the methods described by Guo et al. (2021). The degrading enzymes in the composted substrate were determined by the spectrophotometric methods at 50°C, including protease (Pro), filter paper cellulase (FPase), xylanase (Xyl), and laccase (Lac) (Wang et al., 2012; Zhang et al., 2019).

### Agronomic and nutritional properties

After the harvest, the agronomic properties of mushroom fruiting bodies were determined, including incubation period (IP, time for mycelia fully colonized the substrate), yield of the first flush, total yield of three flushes, biological efficiency (BE), contamination rate (CR), fresh weight of single mushroom (FWSM), and length of stipe (LS), diameter of stipe (DS), thickness of pileus (TP), and diameter of pileus (DP) (Ma N. L. et al., 2020). The BE refers to the amount of fresh fruiting bodies produced from a certain amount of raw materials, and the contamination rate is the proportion of contaminated sticks to total mushroom sticks. They were determined as described by Yang et al. (2022). In detail, BE (%) = the fresh weight of fruiting bodies of each 100 bags harvested from the first three flushes/the dry weight of the relative substrate × 100. CR (%) = number of contaminated bags per 100 cultivation bags. The BE was determined based on the yield of three flushes, whereas the agronomic properties of single mushroom were determined based on the fresh fruiting bodies of the first flush.

The nutritional properties of the first-flush mushrooms were determined, including contents of crude fiber, crude fat, crude protein and amino acids. The fresh fruiting bodies were dried at 60°C in a drying box until a constant weight. The dried samples were subsequently pulverized by an electric crusher, and sifted through a 200-mesh sieve. The obtained powder was used for further assays on nutritional properties. The crude fiber content was determined according to the Chinese National Standard Method (GB/T 5009.10-2003). The crude fat content was determined using a Soxhlet apparatus (GHYK-4A, Gaohuan Youke, China) (Jacinto-Azevedo et al., 2021). The dried mushroom powder was hydrolyzed with HCl (6 mol/L) containing 5 mg/mL of phenol under vacuum at 110°C for 24 h, subsequently analyzed by an automatic amino acid analyzer (L-8800, Hitachi, Japan) (Wang et al., 2022; Guo et al., 2023). The contents of total amino acids (TAA), essential amino acids (EAA), non-essential amino acids (NEAA), sweet amino acids (SAA, Ala, Gly, Pro, Ser and Thr), bitter amino acids (BAA, Arg, His, Ile, Leu, Met and Val), flavour amino acids (FAA, Asp and Glu) and aromatic amino acids (AAA, Phe and Tyr) were further calculated (Liang et al., 2020; Guo et al., 2023).

### High throughput sequencing

The composting samples at the beginning (BM) and ending (CP) stages of CK, P1, P3, and P5 treatments were further analyzed by the high throughput sequencing. The total genomic DNA of composting samples were extracted using a soil DNA kit (E.Z.N.A.^®^, Omega Bio-tek, United States) following the standard protocol. The concentration and quality of DNA obtained were evaluated using a NanoDrop2000 spectrophotometer (Thermo Fisher Scientific, United States) and 1% agarose gel electrophoresis. Subsequently, the high throughput sequencing based on 16S rRNA gene was performed by Majorbio Bio-Pharm Technology Co. Ltd. (China) using a MiSeq PE300 platform (Illumina, United States) (Yang et al., 2022). The primer pairs used for sequencing were 338F/806R for V3–V4 region of 16S rRNA gene. The raw 16S rRNA gene sequence data was further uploaded to the NCBI Sequence Read Archive (SRA) database with the accession number of PRJNA884292.

### Bioinformatics analysis

The raw sequencing data were quality-filtered and assembled into high-quality reads using fastp (v0.20.0) and FLASH (v1.2.7). The operational taxonomic units (OTUs) were picked based on 97% similarity using the high-quality reads and USEARCH (v 7.1). Taxonomy assignment of the 16S rRNA gene sequences was performed using the SILVA database (v138) with the RDP classifier (v2.2) (Yang et al., 2022). The basic analyses were illustrate using the online Majorbio platform^1^ (Guo et al., 2021). The Co-occurrence network analysis was conducted using R software (v4.1.3) with the Spearman’s correlation coefficient (*r*) > 0.6 and statistically significant (*P*) < 0.05 (Barberan et al., 2012). The prediction of bacterial metabolism was performed using the PICRUSt2 tool (v2.2.0) based on the KEGG database. The correlations between physicochemical and bioinformatic parameters were conducted using the Mantel test via online R software^2^ (Yang et al., 2022).

### Statistical analysis

All the data were analyzed using Microsoft Excel (v2016) and Origin (v2020b), and presented as means ± standard deviation (SD). Statistical analysis was performed by one-way ANOVA using SPSS (v.25.0). A *p* < 0.05 was considered statistically significant. The structural equation modeling (SEM) analysis was conducted using SPSS Amos Graphics (v26.0) (Wu et al., 2021).

## Results and discussion

### Physicochemical properties of compost

**Figure 1:**
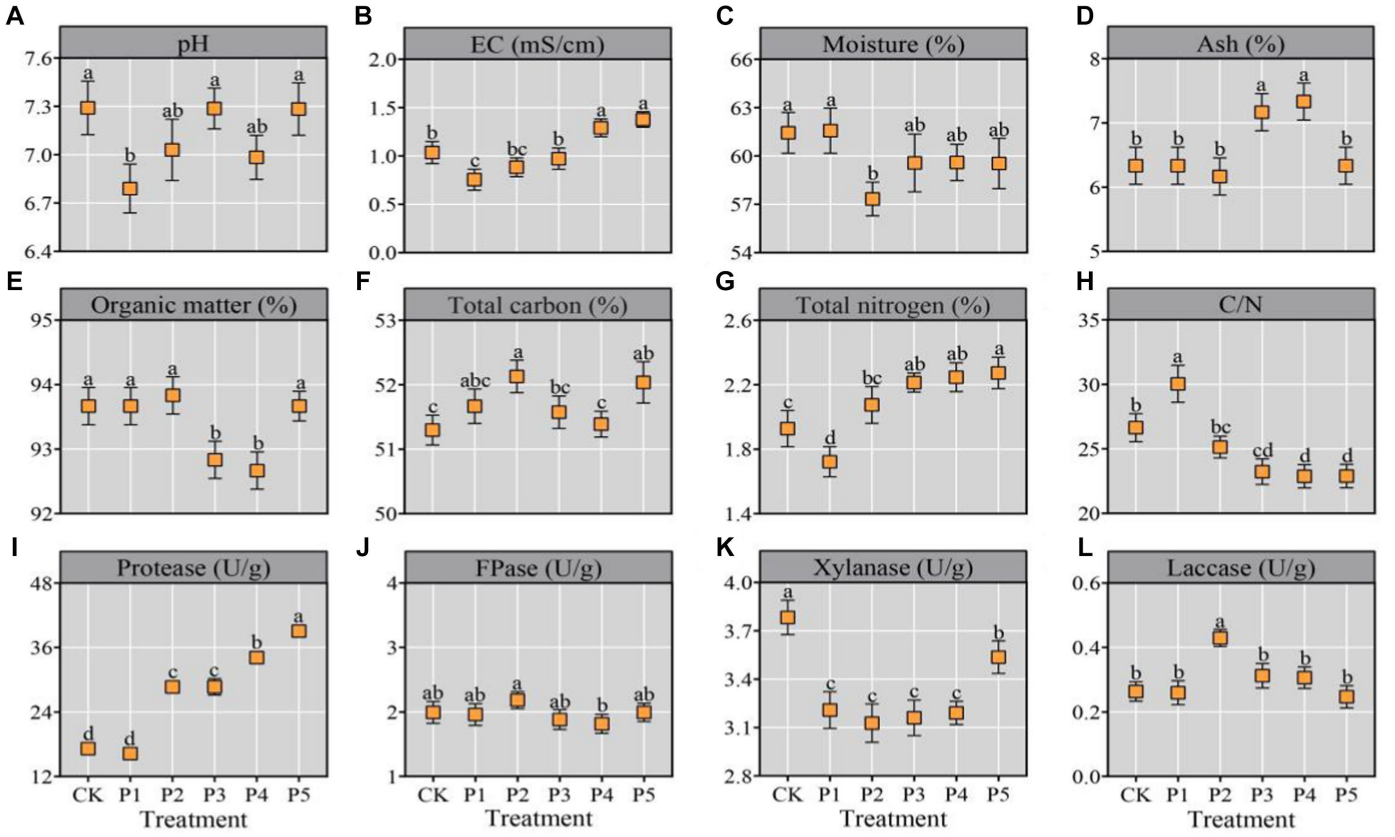
Physicochemical properties of substrate after composting. All the data were expressed as means ± SD (*n* = 3). Different lowercase letters denote significant differences (*p* < 0.05).

The pile temperature of each treatment sharply increased by over 50°C and maintained a thermophilic stage of 51–78°C for two days (Supplementary Figure S1). High temperature promotes the proliferation of thermophilic microorganisms and the decomposition of lignocellulose, which is a critical factor determining the success or failure of composting (Kong et al., 2020; Guo et al., 2021). The physicochemical properties of the composted substrate were shown in Figure 1. All the treatments shared a near-neutral pH range (6.79–7.29) (Figure 1A), which resembles the optimal pH range (6.5–7.0) for mycelial growth of most oyster mushrooms (Wan Mahari et al., 2020). There was a small amount of eggshells in BEM, which ash content was close to that of wheat bran (Supplementary Table S1). With the continuous increase of BEM content from P1 to P5, the EC of compost also continuously increased from 0.76 to 1.38 mS/cm (Figure 1B). Moreover, all the treatments represented relatively low EC, which would not damage the growth of mushroom mycelia (Yang et al., 2022). The P4 and P5 treatments demonstrated significantly (*p* < 0.05) high ash content and low OM content (Figures 1C,D), indicated that the two treatments consumed more OM during composting. Moreover, all the treatments shared a OM range of 92.67–93.87%, which was very close to that of compost after a short-term composting of 4–5 d (Yang et al., 2022).

The TC, TN, and C/N ratio are key factors for oyster cultivition, which directly affect the mycelial growth ratio and mushroom yield (Bellettini et al., 2019). In this study, the TC, TN, and C/N ratio were 51.57–52.13%, 1.72–2.27%, and 23/1–30/1, respectively (Figures 1E–H), which were close to the previous reports (Guo et al., 2021; Yang et al., 2022). Previous reviews on oyster mushroom cultivation indicated a optimun TN range of 1.84–2.08% and an optimum C/N range of 28/1–30/1 (Bellettini et al., 2019; Wan Mahari et al., 2020). Nitrogen participates in many important metabolic pathways in cells, including amino acid, protein and nucleic acid metabolisms. Too low or too high TN will inhibit the mycelial growth of oyster mushrooms and decrease the yield (Wan Mahari et al., 2020). Properly increasing the nitrogen content in the substrate can improve nitrogen metabolism, and thus improve the yield and nutrition properties of oyster mushroom (Rizki and Tamai, 2011; Sözbir et al., 2015). Generally, wheat bran is the main nitrogen source for mushroom cultivation (Bellettini et al., 2019). Eggs are one of the most nutritious foods and perfect protein sources. In this study, the addition of BEM in P3, P4, and P5 treatments significantly (*p* < 0.05) improved the TN in the final compost for mushroom cultivation (Figure 1G). It indicates that the addition of BEM in the composting raw materials may increase the yield and quality of oyster mushroom.

Furthermore, the degrading enzyme activities towards protein and lignocellulose in the compost were determined. With the increase of BEM addition, the protease activity in the compost gradually increased and reached the maximum in the P5 treatment (Figure 1I). It may be due to the addition of BEM activating the composting microorganisms with protease activities. All the six treatments represented a FPase range of 1.81–2.18 U/g, an Xly range of 3.13–3.78 U/g and a Lac range of 0.25–0.43 U/g (Figures 1J–L), which were similar with the degrading enzyme activities in the compost reported by Yang et al. (2022). During the short-term composting processes of substrate for oyster mushroom cultivation, the degradation ratios of cellulose and hemicellulose are usually higher than that of lignin, which was basically consistent with this study (Guo et al., 2021; Yang et al., 2022).

### Agronomic properties of fruiting bodies

The agronomic properties of *P. floridanus* in different treatments were summarized in Table 2. The IP partly reflects the quality of the substrate for oyster mushroom cultivation. The CK, P1, P2, and P3 treatments shared a close IP of 16–19 days, which was approximate to that of *Pleurotus citrinopileatus* mycelia cultivated on the wheat straw substrate (17 days) (Koutrotsios et al., 2022). However, the P4 and P5 treatments represented significantly (*p* < 0.05) longer IP of 32–34 days, indicating that they were not suitable for the mycelial growth of oyster mushroom. They also demonstrated the highest CR of 68.75 and 69.52%, respectively. There may be more miscellaneous microorganisms in the substrates of the two treatments, which inhibited the normal growth of mushroom mycelia. A pasteurization at 100°C for 4 h cannot kill all microorganisms in the compost (Yang et al., 2022). The survived microorganisms during composting process are one of the keys for improving the mushroom yield and reducing the CR (Kong et al., 2020; Guo et al., 2021; Yang et al., 2022). The composting formula, composting time and pasteurization time affect the physicochemical properties and microbial communities of the final substrate for mushroom cultivation (Yang et al., 2022). The addition of high dose of BEM led to higher CR in P4 and P5 treatments. The results indicate that it is necessary to reduce the dosage of BEM or change the composting duration and pasteurization time.

**Table 2:** Agronomic properties of fruiting bodies in different treatments (100 bags each).

Treatment	Incubation period (days)	Contamination rate (%)	Yield of first flush (kg)	Total yield (kg)	Biological efficiency (%)
CK	17 ± 2b	13.30 ± 1.71b	24.40 ± 0.19c	53.21 ± 0.77c	73.91 ± 1.06c
P1	16 ± 2b	1.60 ± 0.27c	28.30 ± 0.46b	64.40 ± 0.49b	89.45 ± 0.68b
P2	17 ± 1b	2.00 ± 0.39c	32.49 ± 0.55a	65.41 ± 0.63b	90.84 ± 0.88b
P3	19 ± 2b	7.00 ± 0.59bc	32.66 ± 0.36a	72.14 ± 0.77a	100.19 ± 1.07a
P4	32 ± 3a	68.75 ± 10.63a	7.22 ± 0.17d	16.41 ± 0.35d	22.79 ± 0.48d
P5	34 ± 3a	69.52 ± 8.61a	6.22 ± 0.15e	14.79 ± 0.24e	20.54 ± 0.33e

All the data were expressed as means ± SD (*n* = 3). Different letters in the same column indicate significant differences (*p* < 0.05).

Oyster mushrooms are efficient lignocellulose decomposers with high yield and BE (Bellettini et al., 2019; Wan Mahari et al., 2020). Many researchers have focused on optimizing the formulas of cultivation substrate to further improve the yield and nutritional quality (Koutrotsios et al., 2022; Wang et al., 2022). In this study, the P3 treatment represented significantly (*p* < 0.05) high yield of the first flush (32.66 kg), total yield (72.14 kg), and BE of three flushes (100.19%) compared with the CK treatment (Table 2). *Pleurotus citrinopileatus* grown on wheat straw, winery and olive mill wastes represented the BE of three flushes of 53.70, 78.52, and 26.24%, respectively (Koutrotsios et al., 2022). *Pleurotus pulmonarius* grown on four kinds of substrates showed a BE range of 61.89–85.01%, based on two flushes (Wang et al., 2022). It suggests that a proper addition of BEM can improve the yield and BE of *P. floridanus*. In addition, the low BE of P4 and P5 treatment was mainly due to the high CR during the IP, resulting in a sharp decline in the total yield. Due to the inhibition of mushroom mycelial growth by miscellaneous microorganisms in the substrate, the utilization of the substrate was reduced, resulting in a decrease in mushroom yield and BE. The agronomic properties of single mushroom were summarized in Supplementary Table S2. Compared with CK, the BEM treatments P1, P2, and P3 were similar in LS, DS, TP, and FWSM (no significant difference). It indicates that a proper addition of BEM as the nitrogen source has no significant effect on the commercial properties of the oyster mushroom.

### Nutritional quality of fruiting bodies

**Figure 2:**
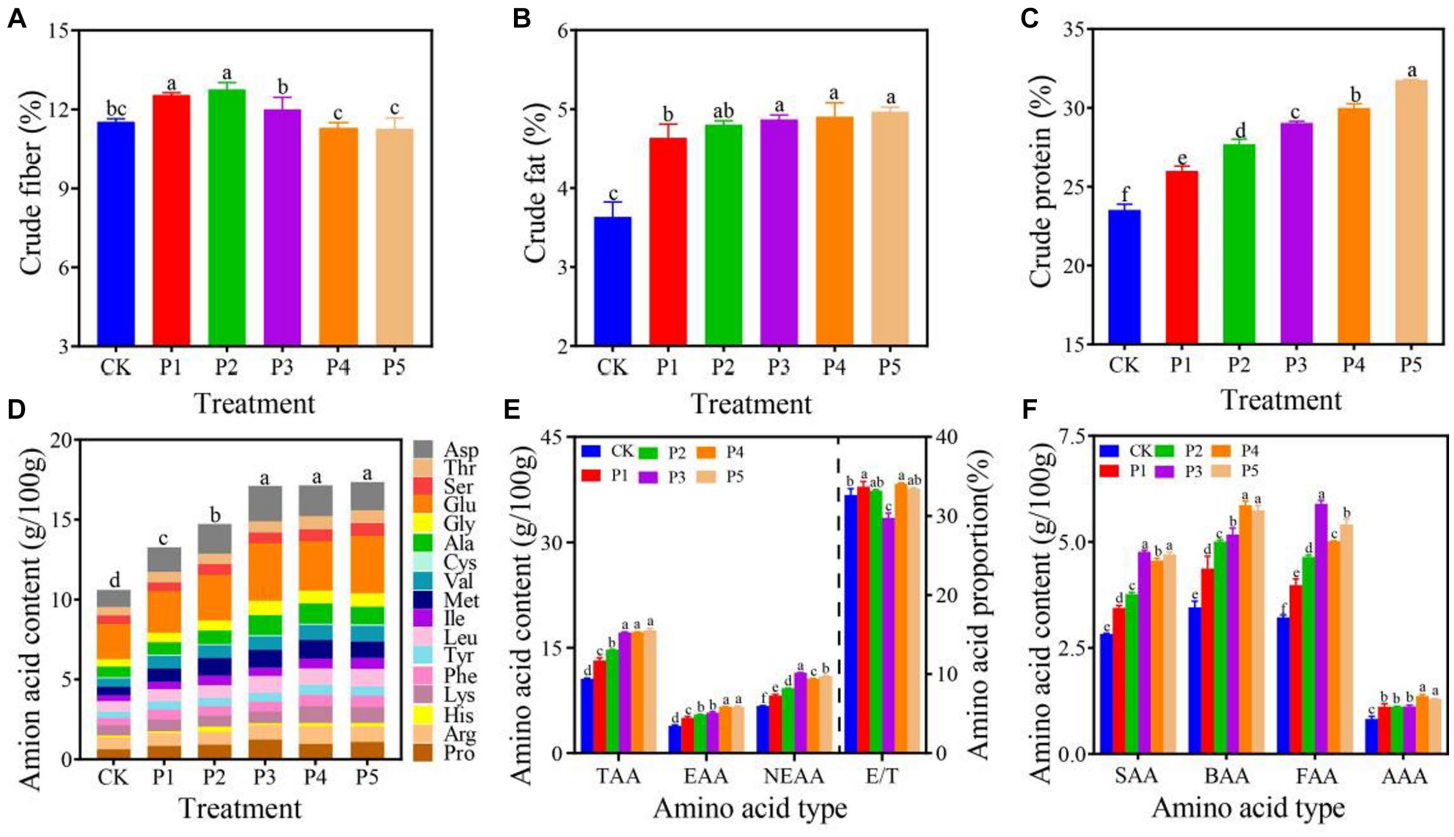
Nutritional composition of *P. floridanus* fruiting bodies grown on diffferent substrates (based on dry weight). AAA, aromatic amino acids; BAA, bitter amino acids; EAA, essential amino acids; E/T, EAA/TAA; FAA, flavour amino acids; NEAA, non-essential amino acids; SAA, sweet amino acids; TAA, total amino acids. All the data were expressed as means ± SD (*n* = 3). Different lowercase letters denote significant differences (*p* < 0.05).

The oyster mushrooms are rich in nutrients, which nutrient composition changes with different cultivation substrates (Suwannarach et al., 2022). Low doses of BEM (P1 and P2 treatments) can significantly (*p* < 0.05) increase the crude fiber content of fruiting bodies, whereas there was no significant (*p* < 0.05) difference between high doses (P3-5) and CK treatments (Figure 2A). It indicates that BEM as the nitrogen source will not reduce the crude fiber content of oyster mushromm fruiting bodies. Furthermore, The addition of BEM significantly (*p* < 0.05) increased the crude fat content of fruiting bodies. The P1–P5 treatments represented a crude fat content range of 4.63–4.96%, which was 27.55–36.64% higher than that of CK (3.63%) (Figure 2B). The crude fat content of mushrooms varies greatly depending on their own characteristics and growth conditions such as nitrogen sources in the substrate (Asaduzzaman Khan and Tania, 2012). *Pleurotus ostreatus* and *P. cystidiosus* showed a crude fat content range of 1.32–2.78% and 2.05–3.33%, respectively, when they were grown on diffferent substrates (Hoa et al., 2015). Eggs contain certain lipids, which led to the significant (*p* < 0.05) increase in crude fat contents in BEM treatments. In addition, lipids are important energy substances and components of cells. Proper increase of the crude fat content can also improve the taste of fruiting bodies.

With the increase of BEM content, the crude protein content of fruiting bodies in BEM treatments increased significantly (*p* < 0.05) from 25.97 to 31.76%, which was 10.46–35.09% higher than that in CK (23.51%) (Figure 2C). The crude protein content of *Pleurotus* genus ranges from 11 to 42% depending on different species and cultivation conditions (Asaduzzaman Khan and Tania, 2012).The crude protein content of *P. floridanus* significantly (*p* < 0.05) increased from 23.3 to 29.8%, when it was grown on rice straw supplemented with *Leucaena leucocephala* foliage (Andrew, 2022). The crude protein content of *P. ostreatus* and *P. cystidiosus* ranged between 19.52–29.70% and 15.68–24.54%, respectively, when they were grown on different substrates (Hoa et al., 2015). Although only P3, P4, and P5 treatments had significantly (*p* < 0.05) higher TN content of substrate than the CK treatment (Figure 1G), the crude protein content of all BEM treatments was significantly (*p* < 0.05) higher than in CK. It indicates that the high content of BEM in substrate contributed to the higher protein content of fruiting bodies.

Furthermore, the amino acid content and taste characteristics of each treatment were determined. The amino acid content of BEM treatments ranged from 13.20 to 17.45 g per 100 g dry weight, which was significantly (*p* < 0.05) higher than that of CK treatment (10.59 g/100 g d.w.) (Figure 2D). The Glu and Asp were the most abundant amino acids in all the treatments, whereas the content of Ala, Arg, Asp. Glu, Gly, Ile, Leu, Met, Pro, Ser, Thr and Val in the BEM treatments were significantly (*p* < 0.05) higher than those in CK (Supplementary Table S3). The Glu and Asp are known as monosodium glutamate like amino acids, which are responsible for the characteristic umami taste of mushrooms (Bellettini et al., 2019; Koutrotsios et al., 2022). It indicates that the friuting bodies grown on BEM additive substrates taste better. Moreover, the addition of BEM also resulted in significantly (*p* < 0.05) higher contents of TAA, EAA, NEAA, SAA, BAA, FAA, and AAA in BEM treatments than in CK, which makes them have higher nutrition and better taste (Figures 2E,F). Compared with the CK treatment, the P3 treatment represented significantly (*p* < 0.05) high TAA, NEAA, SAA, and FAA of 17.17, 11.42, 4.76, and 5.90 g/100 g d.w., respectively. The TAA, SAA, and FAA contents of *P. pulmonarius* ranged between 18.90–27.97, 12.25–19.35, 0.35–1.12, and 0.69–1.16 g/100 g d.w., respectively grown on three different substrates and the first two flushes (Wang et al., 2022). In addition, the EAA/TAA (E/T) range of all treatment was 33.57–37.40%, which was close to that of *P. pulmonarius* (31.00–37.00%) and other edible wild-grown mushrooms from China (30.50–43.40%) (Wang et al., 2014, 2022). In brief, the BEM is a high-quality nitrogen source for oyster mushroom cultivation, which can improve the crude protein content, amino acid composition and flavor of the fruiting bodies.

### Bacterial communities in the substrate

**Figure 3:**
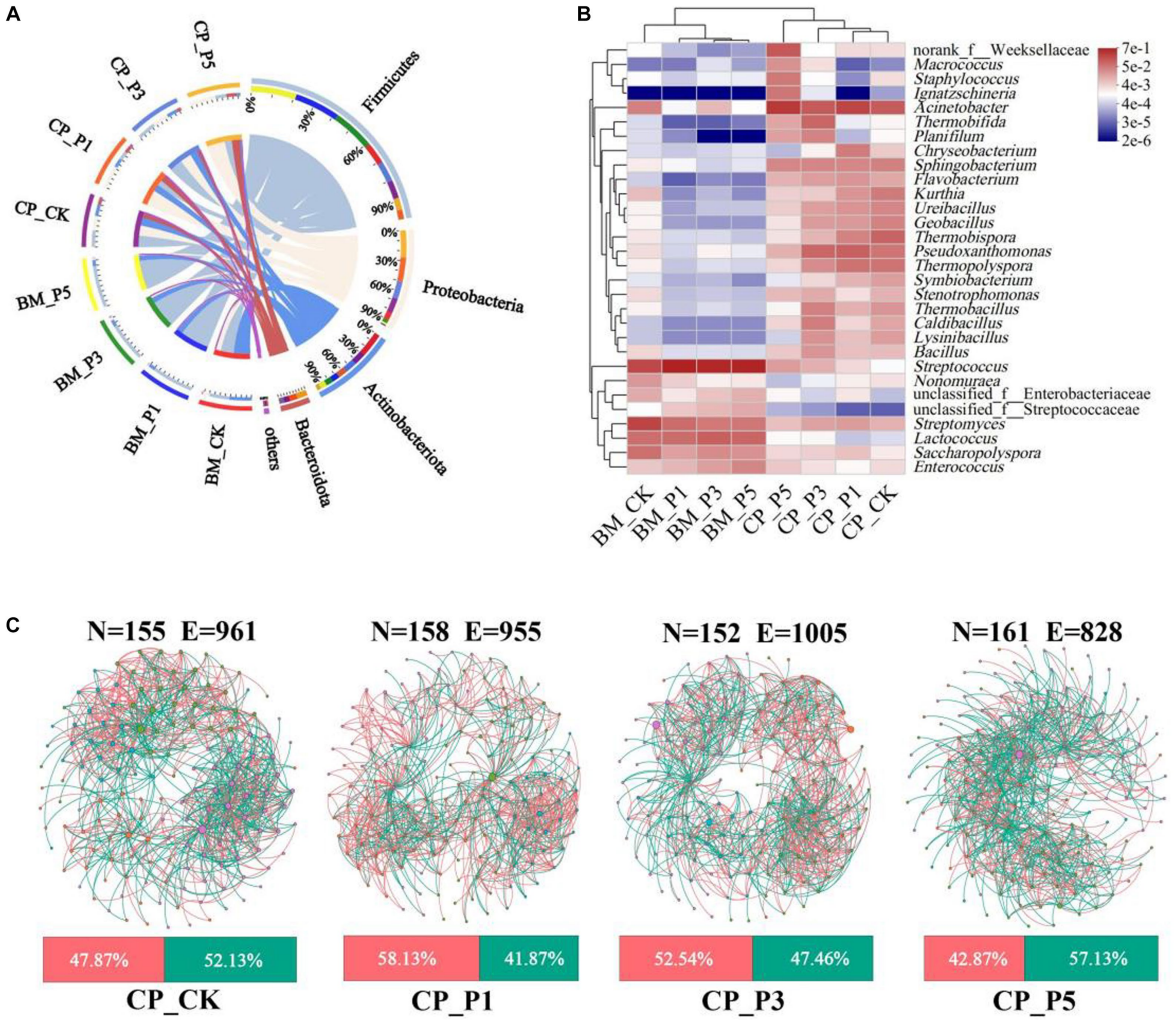
Bacterial composition and correlation in substrate samples. **(A)** Circos diagram at phylum level. Phyla with relative abundance <5% were combined together and indicated as “others.” **(B)** Heatmap of log relative abundance of top 30 genera. **(C)** Co-occurrence network patterns on genus level (top 200 genera) in CP treatments (*r* > 0.6, *p* < 0.05). N, node; E, edge. The pink and green edges depict positive and negative correlations, respectively. The nodes represent individual genera, and node size corresponds to their relative abundance.

Thermophilic bacteria are reported to play important roles in the composting process for oyster mushroom cultivation substrates (Yang et al., 2022). To further evaluate the impacts of composting process on the agronomic and nutritional properties of fruiting bodies, the high-throughput sequencing was performed based on the substrate of CK, P1, P3, and P5 treatments at BM and CP stages. A total of 23 phyla, 59 classes, 152 orders, 259 families, 535 genera, and 1,204 OTUs of bacteria obtained with a sequence similarity ≥97% (Supplementary Table S4). Actinobacteriota (44.77%), Firmicutes (39.42%), and Proteobacteria (13.44%) were the predominant bacterial phyla in BM_CK, whereas the predominant bacterial phyla of BEM treatments at BM stage were Firmicutes (77.61–82.90%), Actinobacteriota (12.41–13.80%) and Proteobacteria (2.34–6.71%) (Figure 3A). These phyla accounted for 97.64–99.00% of the entire representative of bacterial sequences in the samples. It indicates that almost all bacteria in substrates were detected by the high-throughput sequencing (Qiu et al., 2022). The predominant bacterial phyla of BM_CK were similar with those of samples before composting for oyster mushroom cultivation (Guo et al., 2021). The addtion of BEM strongly increased the relative abundance of Firmicutes speciese at BM stage, which could be attributed to the bacterial community of BEM itself. After the short-term composting process, Firmicutes (33.01%), Proteobacteria (28.70%), Actinobacteriota (22.77%), and Bacteroidota (11.37%) were the predominant bacterial phyla in CP_CK, whereas Proteobacteria (32.03–50.03%), Firmicutes (17.32–37.34%), and Actinobacteriota (6.67–21.18%) became the predominant bacterial phyla in BEM treatments. After composting, the predominant bacterial phyla in CK and BEM treatments tended to be similar. Firmicutes, Actinobacteriota, Proteobacteria were the most abundant bactelial phyla in the composted substrate of peach sawdust-based formulas (Guo et al., 2021; Yang et al., 2022). Proteobacteria and Firmicutes were reported to be the dominant phyla in the sugarcane straw-based substrate during a 5–15 days’ composting for oyster mushroom cultivation (Vieira et al., 2019). They play important roles in the lignocellulosic decomposition and nitrogen conversion in the thermophilic stages during composting (Liu et al., 2022).

Furthermore, the heatmap analysis of top 30 genera among all samples were performed (Figure 3B). *Streptomyces* (26.32%) and *Streptococcus* (24.05%) were the most prevalent genera in BM_CK, while *Streptococcus* (59.29–70.56%) and *Lactococcus* (8.57–12.51%) were predominant genera in BEM treatments at BM stage. Guo et al. (2021) reported that *Streptomyces* was the top abundant genus (24.0%) in the substrate at BM stage, which came from the natural composting of the raw materials. The high abundance of *Streptococcus* and *Lactococcus* in the BEM treatments presumably came from the production and storage processes of BEM. After composting, the relative abundance of *Streptomyces* and *Streptococcus* in the four CP treatments decreased dramatically to 0.86–2.33% and 0.13–0.43%, respectively, while *Acinetobacter* became the predominant genus 14.20–35.79%. *Acinetobacter* was the most abundant genus in the thermophilic stage during the corncob-based and sugarcane straw-based composting, and contributed to the lignocellulosic degradation (Vieira et al., 2019). Moreover, *Thermobifida* (10.53%) and *Pseudoxanthomonas* (10.20%) became the predominant genera in CP_P3 treatment. They were thermo-tolerant and involved in carbohydrate and nitrogen metabolism (Kong et al., 2020).

### Alpha and beta diversity of bacterial communities

The richness and diversity of bacterial communities can be evaluated using alpha diversity indices (Qiu et al., 2022). The coverage of each sample was ≥0.99, which indicated that the sequencing results included the majority of bacteria in the samples (Supplementary Table S5). The ACE and Chao 1 indexes of BM samples were higher than those of CP samples, suggesting that there were more OTUs in BM samples. Compared with BM treatment, the shannon index was increased and the simpson index was decreased in the CP treatments. The changes of the shannon and simpson indexes indicated that the bacterial communities increased after composting (Qiu et al., 2022).

The PCoA based on the Bray-Curtis distance can visualize the differences in bacterial community composition. The PCoA of bacterial communities in samples at OTU level was showed in Supplementary Figure S2. Three BEM treatments were clustered before composting, whereas CP_CK, CP_P1, and CP_P3 were clustered at the end of composting. However, the bacterial community of CP_P5 treatment was away from the cluster of other treatments. It indicates that CP_P5 treatment required longer composting duration, which also indirectly explained the high CR and low yield in the cultivation stage of the treatment.

### Co-occurrence network analysis of bacterial communities

To further evaluate the correlations of microorganisms in the composted substrate, four co-occurrence networks were constructed with the top 200 bacterial genera (Figure 3C), and the main topological properties of the networks were summarized in Supplementary Figure S3. The power law (*R*^2^) of 0.7885, 0.7994, 0.8201, and 0.8194 were recorded in all the networks of CP_CK, CP_P1, CP_P3, and CP_P5, respectively, indicating the non-random pattern and scale-free nature of the networks (Zhu et al., 2021). The P3 treatment demonstrated the least nodes (152), the most edges (1,005, 52.54% positive) and the highest average degree (13.22) and average clustering coefficient (0.56). This indicates that the P3 treatment enhanced the network complexity of bacterial community. With the increase of BEM dosage, the positive interaction ratio of the three BEM treatments continuously decreased, indicating that the increase of BEM dosage may promote the competition of composting microorganisms for nutrients (Bello et al., 2020; Zhu et al., 2021).

### Heatmap analysis

**Figure 4:**
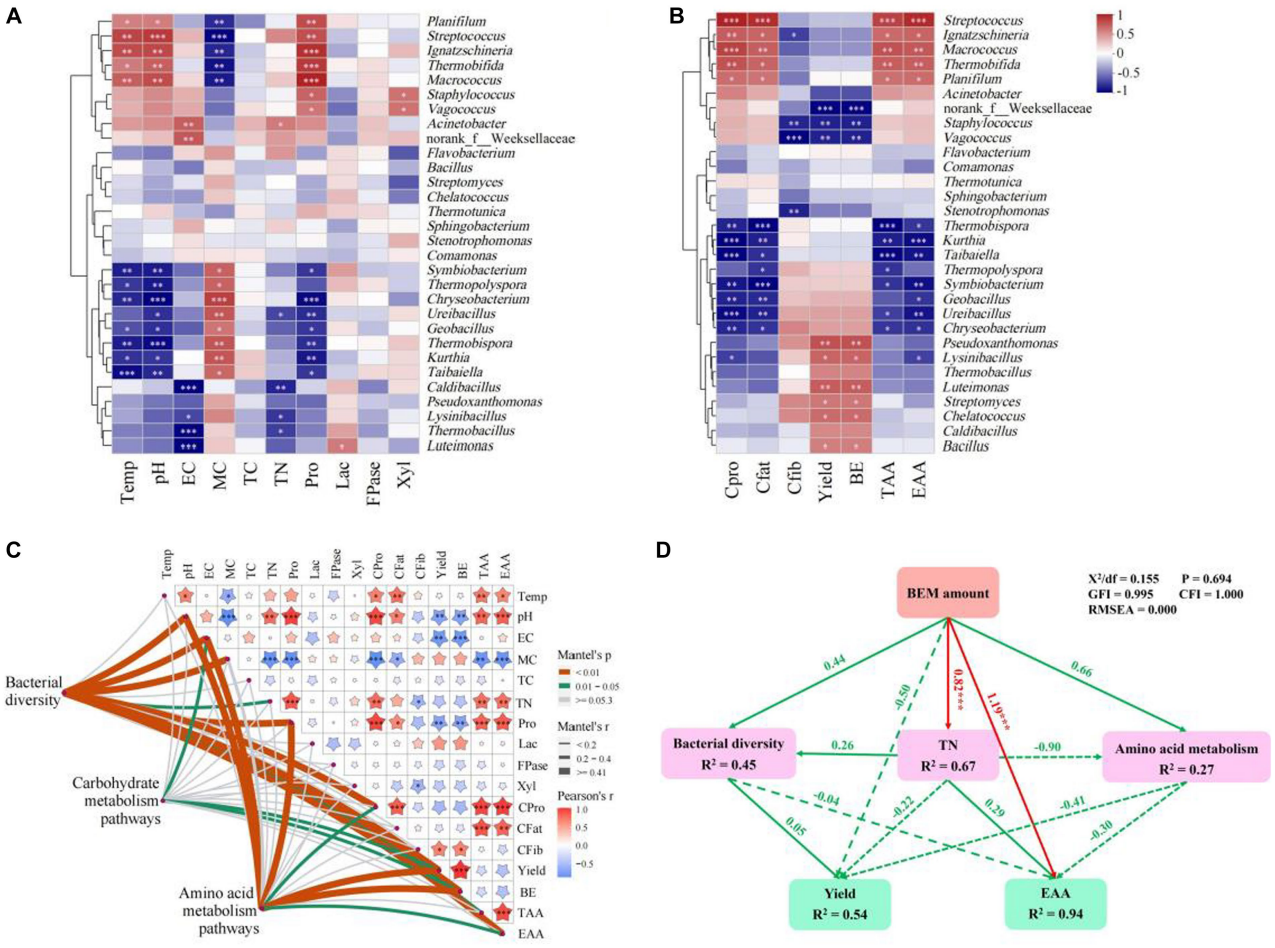
Correlation analysis of bacterial communities of substrate at CP stage with physicochemical, agronomic and nutritional properties. The correlation heatmap of the top 30 genera with physicochemical properties of substrate at CP stage **(A)** and agronomic and nutritional properties of fruiting bodies **(B)**. **(C)** Mantel test based on the Spearman’s correlation coefficients. **(D)** Structural equation model. The red and green arrows represent significant and non-significant relationships, respectively. Temp, temperature; EC, electrical conductivity; MC, moisture content; TC, total carbon; TN, total nitrogen; Pro, protease; Lac, laccase; FPase, filter paper cellulase; Xyl, xylanase; Cpro, crude protein content; Cfat, crude fat content; Cfib, crude fiber content; BE, biological efficiency; TAA, total amino acids; EAA, essential amino acids. *0.01 ≤ *p* < 0.05, **0.001 ≤ *p* < 0.01, and ***p < 0.001.

The bacterial communities and physicochemical properties in the substrate at CP stage play vital roles in the yield and quality of oyster mushroom by the composting cultivation method (Guo et al., 2021; Yang et al., 2022). The heatmap analysis was constructed based on the top 30 bacterial genera at CP stage, physicochemical properties of substrate and agronomic and nutritional properties of fruiting bodies (Figures 4A,B). *Acinetobacter*, the predominant genus at CP stage, represented significant positive correlations with EC (*p* < 0.01) and TN (*p* < 0.01). Previous studies reveal that *Acinetobacter* is predominant in the thermophilic stage and actives in lignocellulosic degradation (Vieira et al., 2019). Moreover, *Thermobifida* showed significant positive correlations with composting temperature (*p* < 0.05), pH (*p* < 0.01), protease activity (*p* < 0.001), crude protein content (*p* < 0.01), crude fat content (*p* < 0.05), TAA (*p* < 0.01), and EAA (*p* < 0.01), whereas *Pseudoxanthomonas* demonstrated significant positive correlations with yield (*p* < 0.01) and BE (*p* < 0.01). *Thermobifida* is thermostable and can participate in cellulose degradation (Liu et al., 2022). In this study, the genus also participated in nitrogen metabolism of the substrate, and improved the nutritional properties of mushrooms. Although the relative abundance of *Streptococcus* declined sharply at CP stage, it was significantly positively correlated with compost temperature (*p* < 0.01), pH (*p* < 0.001), protease activity (*p* < 0.01), crude protein content (*p* < 0.001), crude fat content (*p* < 0.001), TAA (*p* < 0.001), and EAA (*p* < 0.001). It is worth mentioning that many genera represented significant (*p* < 0.05) correlations with compost temperature, pH, MC and protease activity of the composted substrate, and crude protein content, crude fat content, TAA, EAA, yield and BE of fruiting bodies. It indicates that the addition of BEM mainly affected the nitrogen metabolism during composting and mushroom cultivation.

### Functional prediction of bacterial communities

The metabolic properties of bacterial communities in the composted substrate (CP stage) were predicted by PICRUSt based on the KEGG database and visualized in Supplementary Figure S4. The majority of predicted functional genes were assigned into metabolism (40.36–40.77%), genetic information processing (GIP, 6.78–7.24%), environmental information processing (EIP, 5.93–6.67%), and cellular processes (CP, 2.50–3.72%). Eleven pathways were observed for metabolism, three for GIP, two for EIP and two for CP on KEGG pathway level 2. It was consistent with previous reports of composting processes for oyster mushroom cultivation (Guo et al., 2021; Yang et al., 2022). The carbohydrate metabolism (9.31–10.15%), amino acid metabolism (8.54–8.74%), energy metabolism (4.58–4.78%), and metabolism of cofactors and vitamins (4.47–4.62%) were the most predominant pathways on KEGG pathway level 2 (Supplementary Figure S4A). This indicates that the predicted genes involved in carbohydrate and amino acid metabolism were the most abundant at CP stage. A metagenomics sequencing on a corncob-based composting cultivation process revealed that carbohydrate, amino acid and energy metabolism were the top abundant pathways at the end of composting process (Liu et al., 2022). Moreover, the P5 treatment demonstrate significantly (*p* < 0.05) low abundances of carbohydrate metabolism and metabolism of other amino acids, but significantly (*p* < 0.05) high abundance of amino acid metabolism, metabolism of cofactors and vitamins, lipid and nucleotide metabolism. This may indicate that the composting process of P5 treatment was not completed at the end of composting stage, resulting in high CR and low yield of fruiting bodies.

### Mantel test analysis

The correlations among bacterial communities and the matrixes of physicochemical, agronomic and nutritional properties were constructed using the Mantel test (Figure 4C). Pairwise comparisons of physicochemical, agronomic and nutritional properties revealed that temperature, pH, EC, MC, TN and protease activity were the key factors at CP stage, which would further significantly (*p* < 0.05) affect crude protein content, crude fat content, yield, BE, TAA and EAA of mushroom fruiting bodies. A composting duration study reported that pH, EC and TN were the key factors during the thermophilic stage and further affected the yield of oyster mushroom (Yang et al., 2022). The Mantel test analysis revealed that pH, EC, MC, TN and protease activity of the composted substrate significantly (*p* < 0.05) affected the bacterial communities (bacterial diversity at OTU level), while the bacterial communities in the substrate significantly (*p* < 0.05) affected the yield, BE, TAA and EAA of oyster mushrooms. The carbohydrate metabolism pathways significantly (*p* < 0.05) correlated with EC of substrate, and yield and TAA of mushrooms, whereas amino acid metabolism pathways significantly (*p* < 0.05) correlated with pH, EC and protease activity of substrate, and crude protein content, BE and EAA of mushrooms. Although the research on composting processes of agricultural wastes has been widely carried out, the research on short-term composting processes for mushroom cultivation is comparatively limited especially the impact of composting processes on the nutritional quality of mushrooms (Liu et al., 2022; Yang et al., 2022). Guo et al. (2021) reported that TN, temperature and lignin content were the key factors for composting maturity of a short-term peach sawdust-based composting. The C/N ratio, pH, temperature and organic matters significantly affected the succession of microbiota in compost of corncob-based composting (Kong et al., 2020). In this study, the addition of BEM changed the TN, pH, EC, MC and protease activity in the substrate, which determined the bacterial communities in the compost. The bacterial communities further affected the agronomic and nutritional properties of mushroom fruiting bodies.

### Structural equation model analysis

The core objective of this study was to evaluate the impacts of BEM addition on bacterial communities in the substrate, and agronomic and nutritional properties of oyster mushroom. Therefore, the SEM was constructed to further clarify the effects of BEM amount on the short-term composting and oyster mushroom cultivation (Figure 4D). The hypothetical models fit the data well with the parameters of *X*^2^/df = 0.155, *p* = 0.694, GFI = 0.995, CFI = 1.000, and RMSEA = 0.000 (Wu et al., 2021). The BEM amount strongly positively affected the TN (*λ* = 0.82, *p* < 0.001) and EAA (*λ* = 1.19, *p* < 0.001). This indicates that the addition of BEM in the substrate significantly affects the TN of the substrate and nutritional quality of mushrooms. In addition, the effects of BEM amount on bacterial diversity, amino acid metabolism and yield were insignificant. This may be due to the differences of bacterial communities between P5 and other treatments. The high BEM amount in P5 treatment led to the immature substrate, which led to the lowest yield and the highest CR of mushrooms.

## Conclusion

In summary, the BEM is a high-quality alternative nitrogen source for the cultivation of oyster mushrooms using the composted substrate. A proper addition of BEM can improve the physical and chemical properties of the substrate at the end of the composting stage, thus increase the yield, BE, crude fat content, crude protein content, TAA, EAA, NEAA, SAA, BAA, FAA, and AAA of the fruiting bodies. The high throughput sequencing revealed that the addition of BEM significantly changed the bacterial communities in the substrate at the beginning of composting, while those in the mature substrate tended to be similar at the end of composting. The physicochemical properties of substrate significantly affected bacterial communities, which further affected agronomic and nutritional properties of oyster mushrooms. This indicates that BEM can be appropriately added during the composting cultivation of oyster mushrooms to improve yield and quality. These findings reveal the effect of short-time composting on the nutritional quality of oyster mushrooms, and provide a new method for high-quality and efficient use of broken eggs in poultry industry.

## Data availability statement

The original contributions presented in the study are publicly available. This data can be found here: PRJNA884292.

## Author contributions

JW: Conceptualization, Data curation, Supervision, Writing – original draft, Writing – review & editing. Y-XW: Conceptualization, Data curation, Investigation, Software, Writing – original draft. T-KG: Data curation, Investigation, Writing – original draft. Q-YW: Data curation, Investigation, Writing – original draft. JZ: Data curation, Investigation, Writing – original draft. J-YZ: Data curation, Investigation, Writing – original draft. J-LW: Data curation, Investigation, Writing – original draft. Q-JC: Methodology, Writing – original draft. G-QZ: Formal analysis, Funding acquisition, Supervision, Writing – original draft.

## Footnotes

^1^www.i-sanger.com

^2^https://www.cloudtutu.com/#/manteltest
