# Supplementary material for: Bacterial communities during composting cultivation of oyster mushroom Pleurotus floridanus using broken eggs as the nitrogen source and study of agronomic and nutritional properties
Source: Front Microbiol. 2024 Jan 12;14:1274032. doi: 10.3389/fmicb.2023.1274032 (PMC10822690; doi:10.3389/fmicb.2023.1274032)

**The result for leave-one-out analysis**

class Actinobacteria class Negativicutes


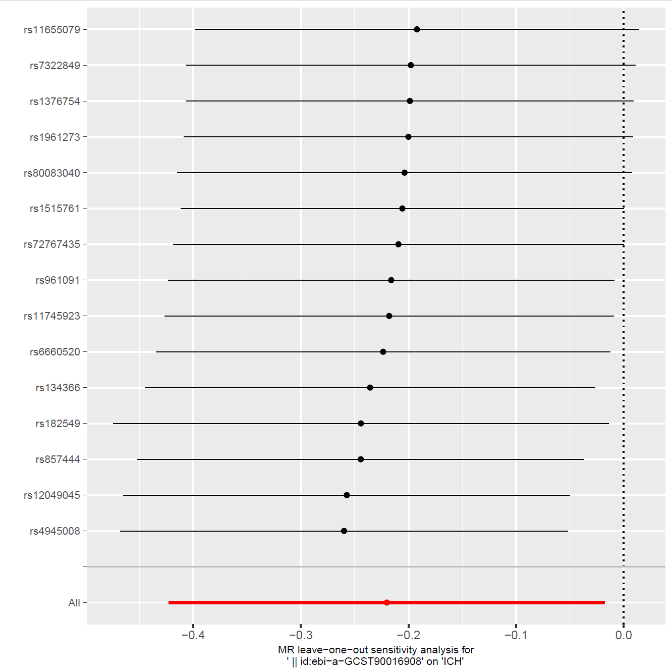

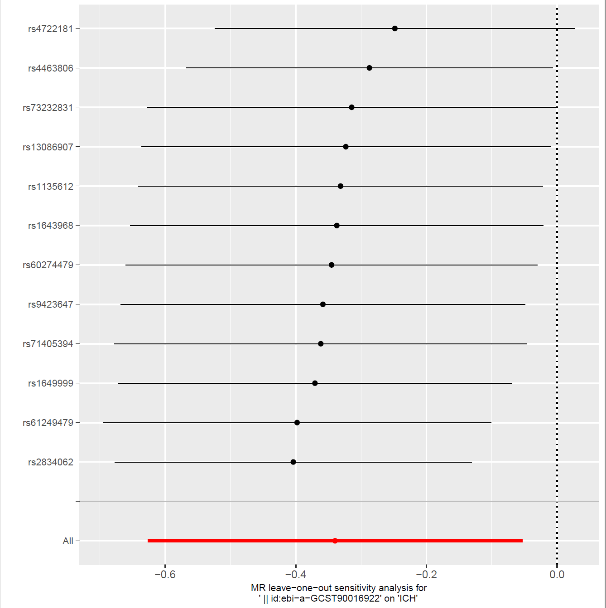


genus Dorea genus Eubacterium eligens


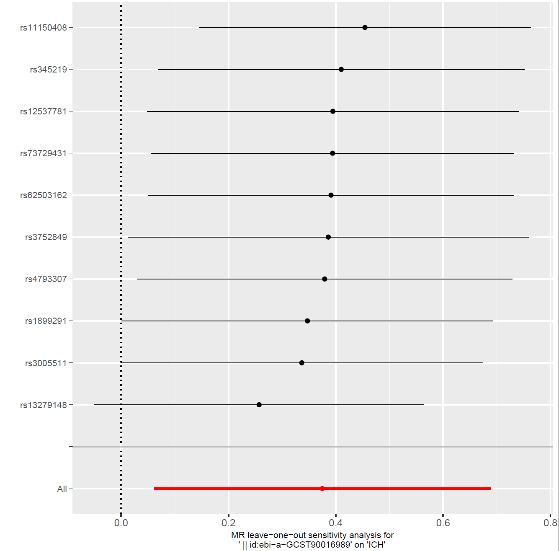

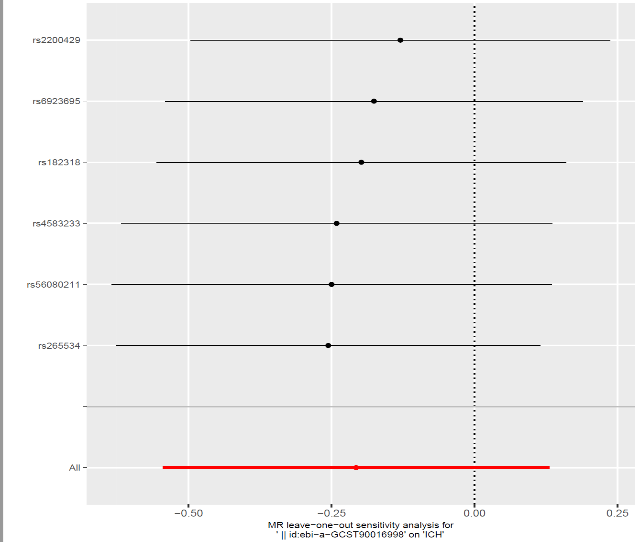


genus Eubacterium rectale genus Eubacterium xylanophilum


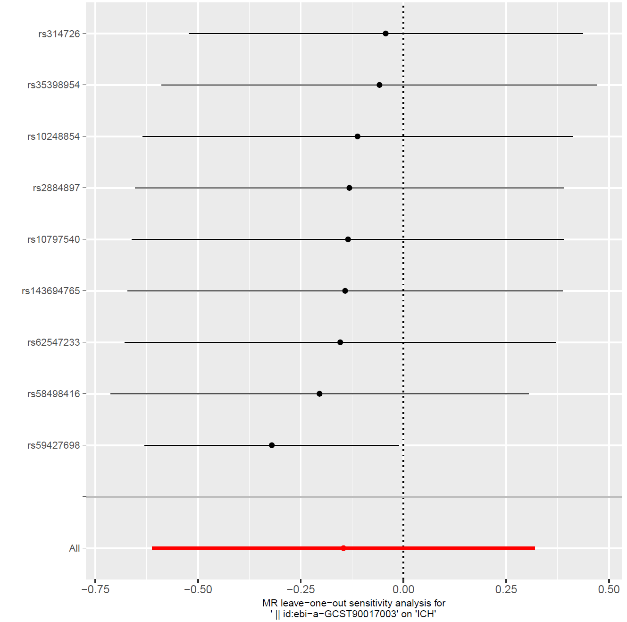

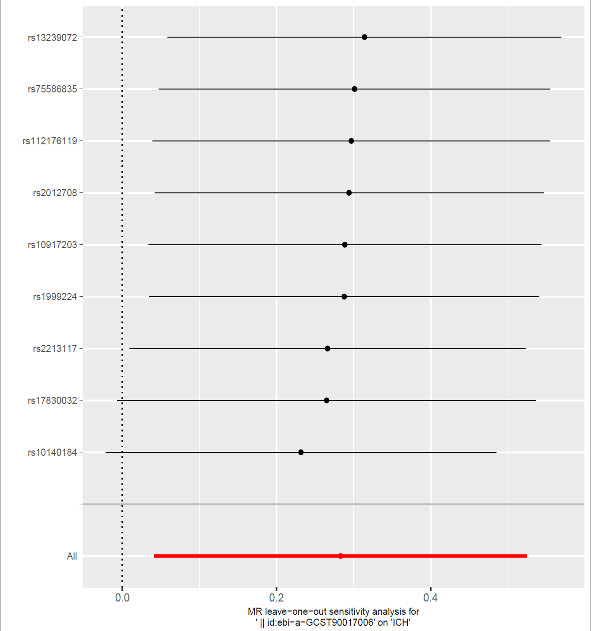


genus Lachnospiraceae ND3007 genus Lachnospiraceae UCG001


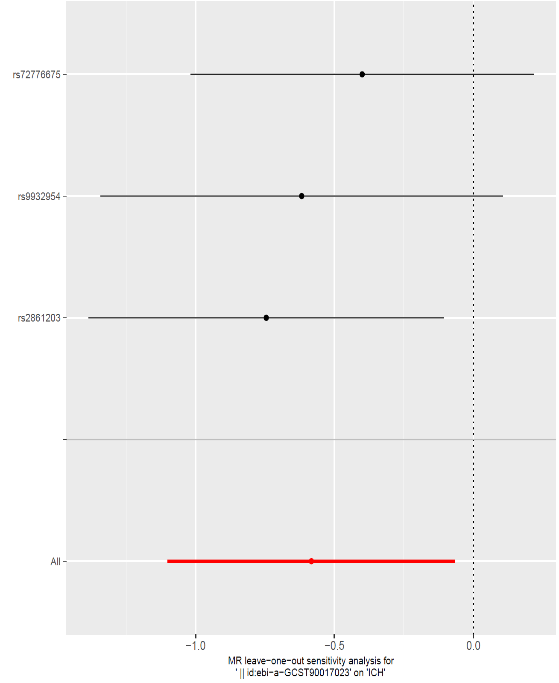

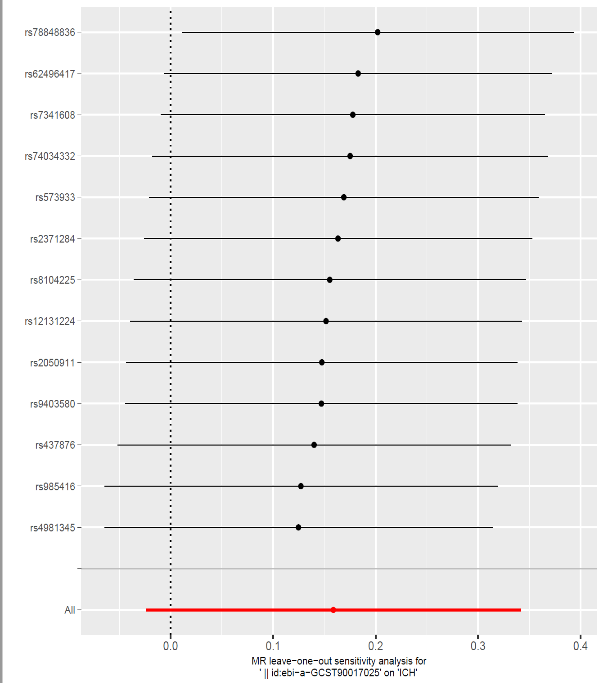


genus Ruminococcaceae UCG009 genus Ruminococcaceae UCG011


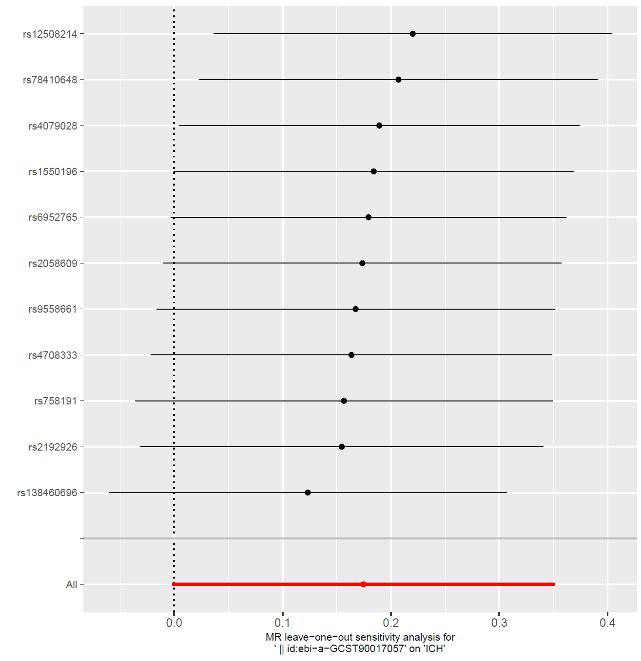

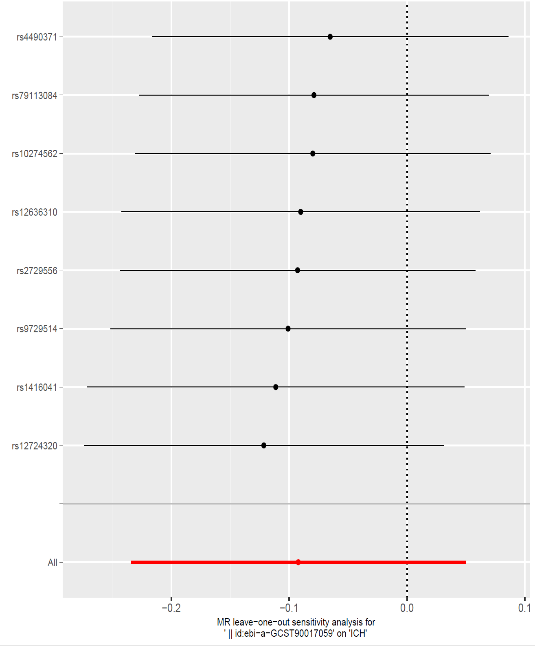


genus Ruminococcus2 genus Senegalimassilia


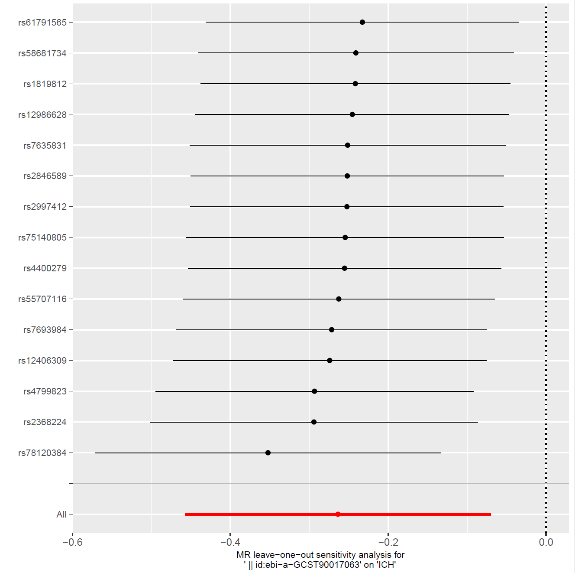

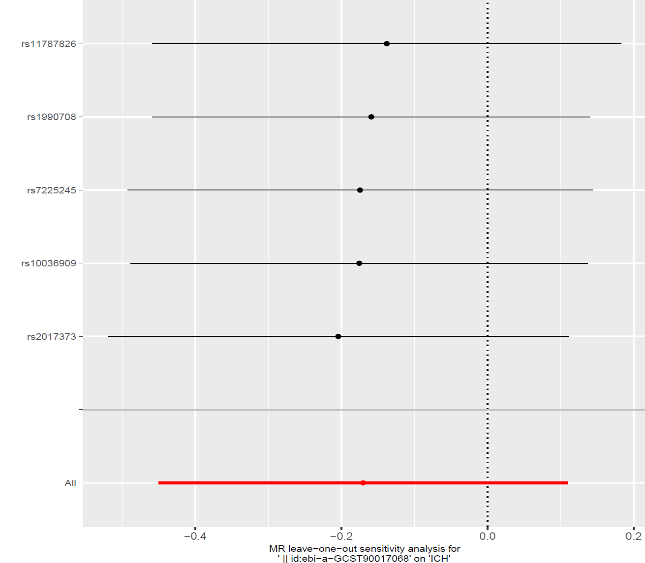


order Mollicutes RF9


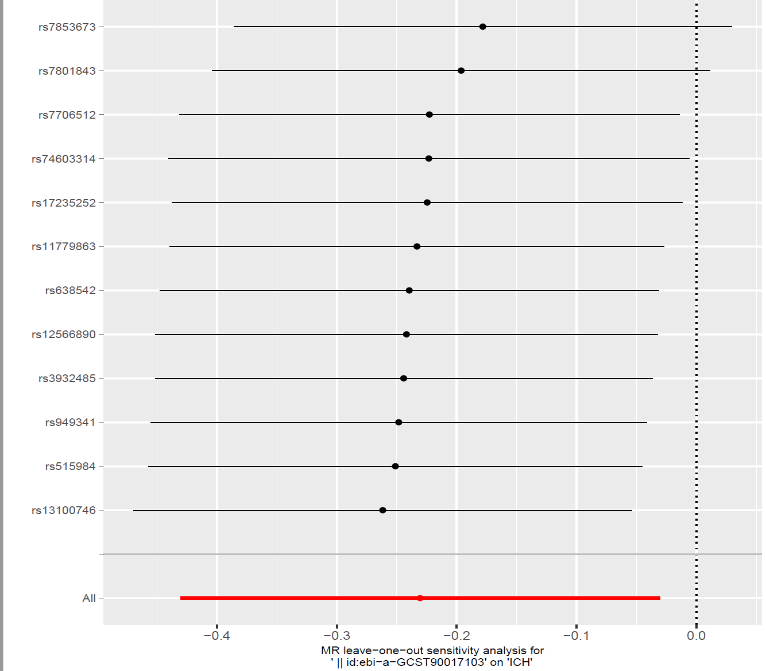


order Selenomonadales


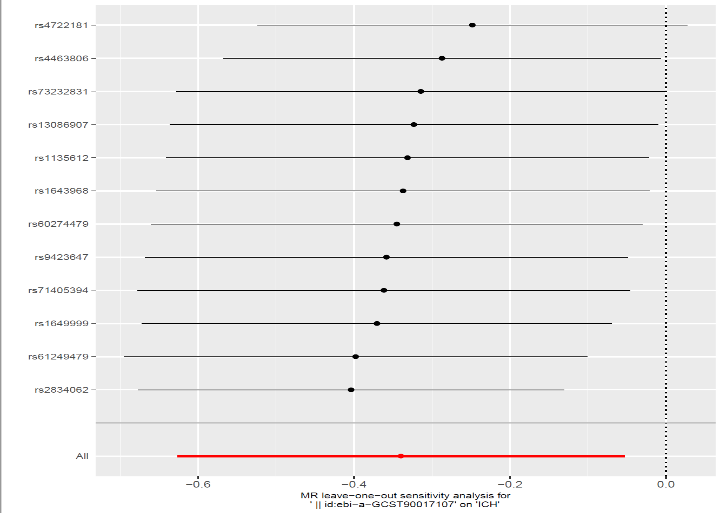


phylum Actinobacteria


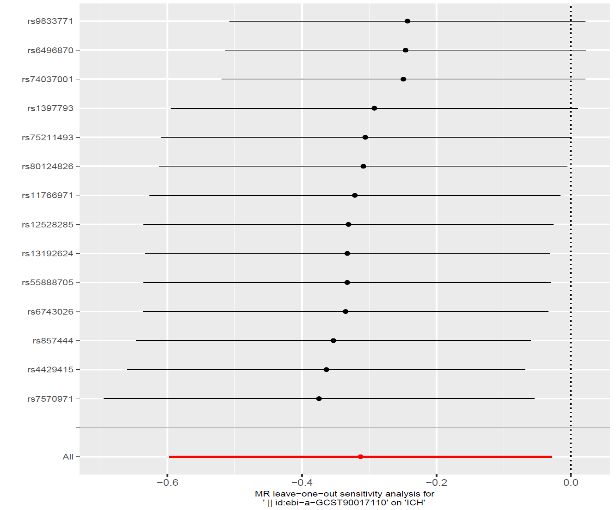


Mannose Uridine


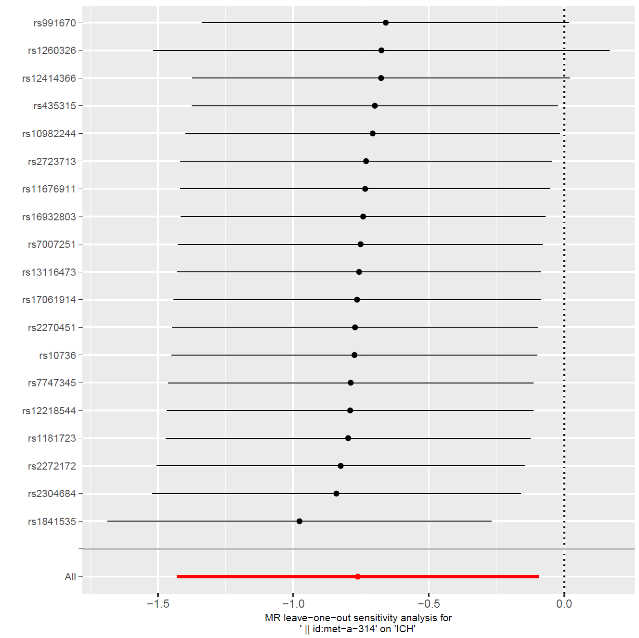

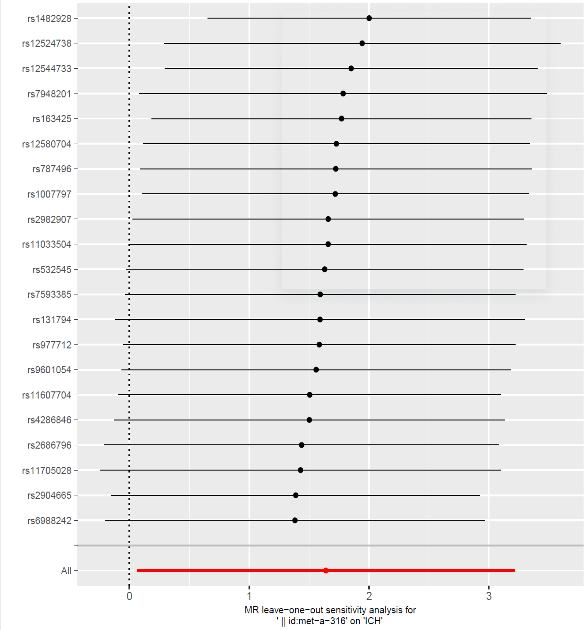


Myristate Phenylacetate


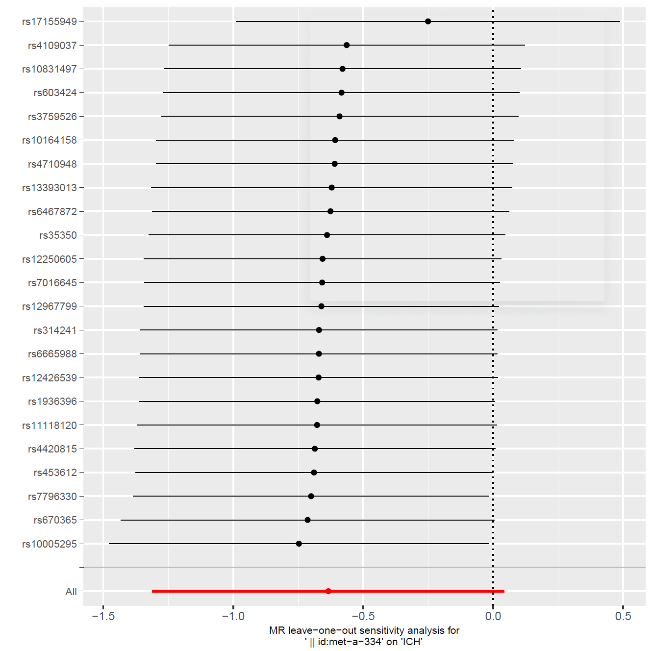

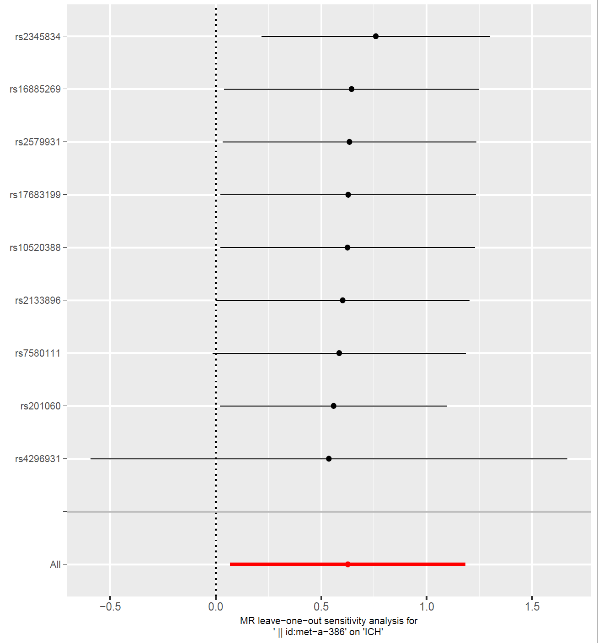


Pyroglutamylglycine Bilirubin


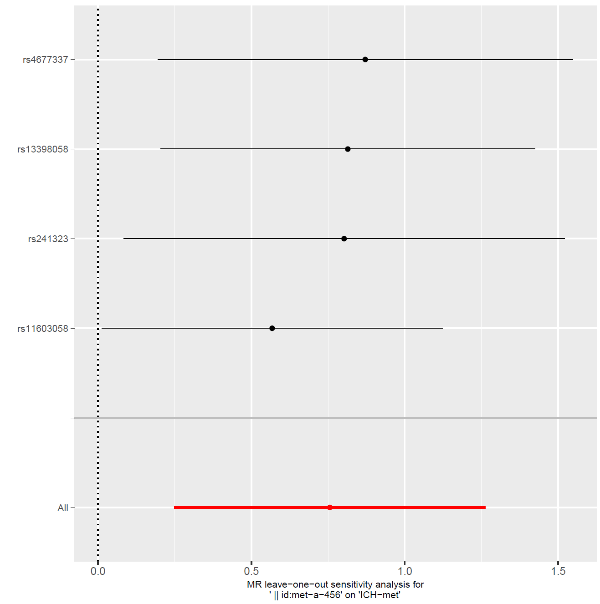

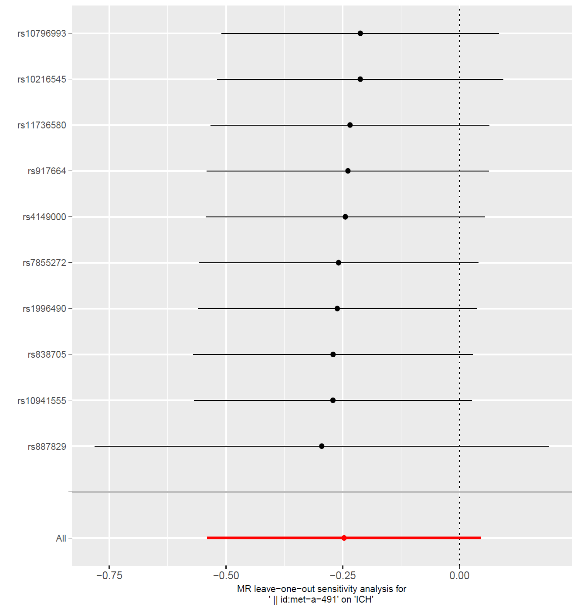


Decanoylcarnitine 1-arachidonoylglycerophosphoinositol


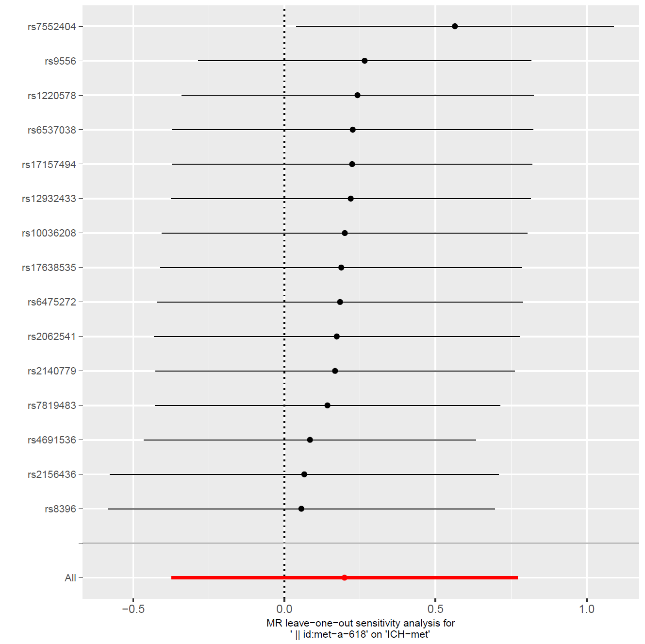

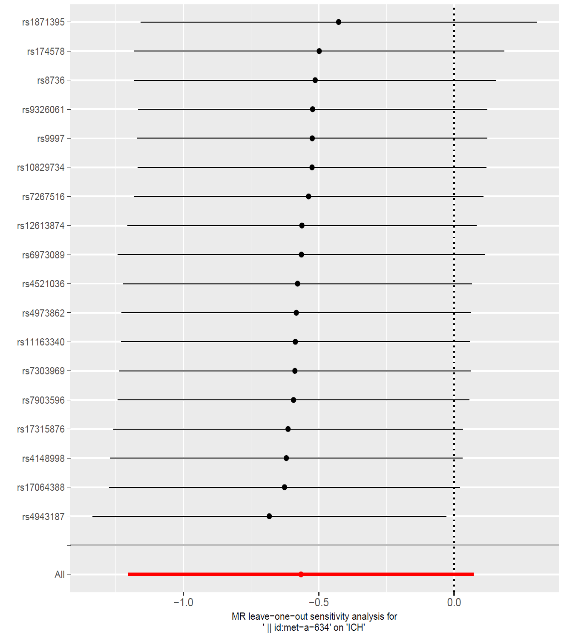


Isovalerylcarnitine


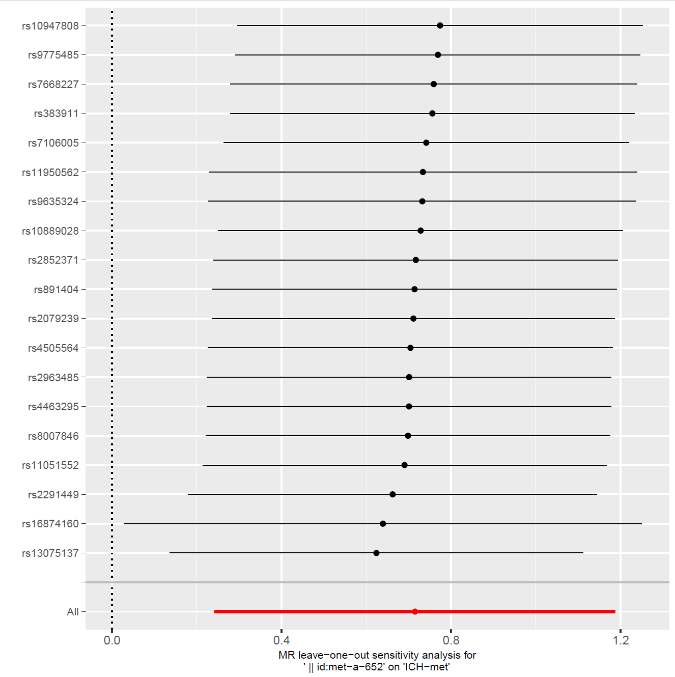


Isovalerate


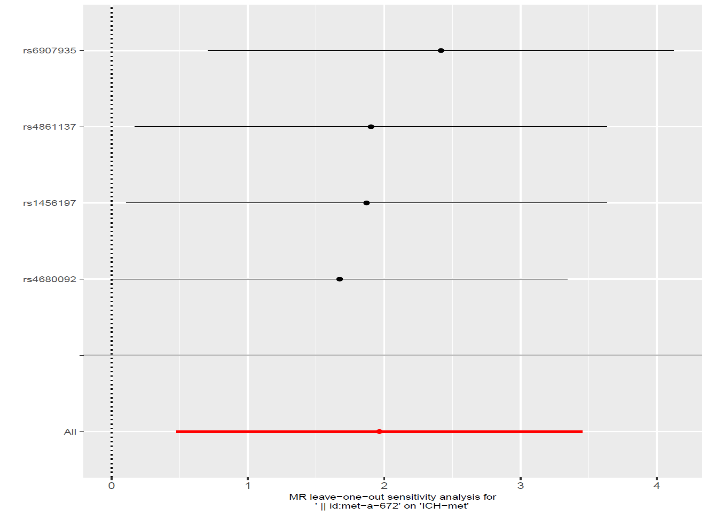


Cysteine-glutathione disulfide


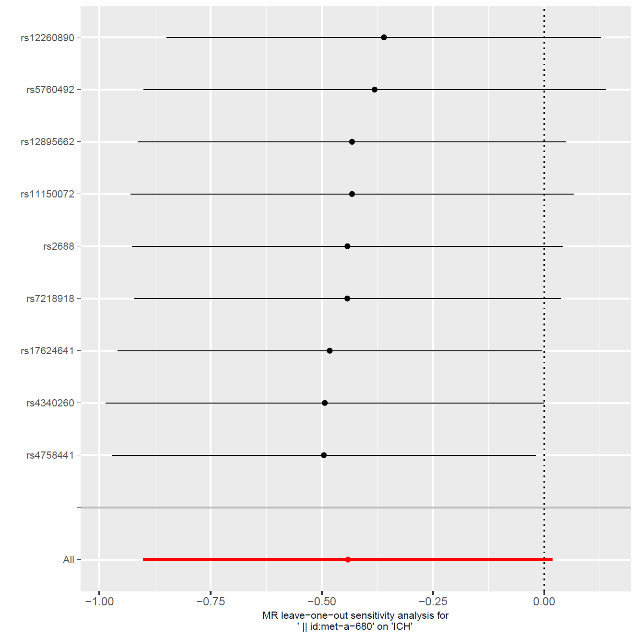

Supplement: Supplementary file 1 [file Data_Sheet_1.docx]
